# Genome-Wide Identification and Transcriptional Analysis of *AP2/ERF* Gene Family in Pearl Millet (*Pennisetum glaucum*)

**DOI:** 10.3390/ijms25052470

**Published:** 2024-02-20

**Authors:** Liang Xu, Ying Lan, Miaohong Lin, Hongkai Zhou, Sheng Ying, Miao Chen

**Affiliations:** 1 College of Agricultural Sciences, Guangdong Ocean University, Zhanjiang 524091, China; xliang2023@163.com (L.X.);; 2 Department of Biochemistry and Molecular Biology, Michigan State University, East Lansing, MI 48823, USA; 3 Shenzhen Institute, Guangdong Ocean University, Shenzhen 518120, China

**Keywords:** abiotic stresses, dehydration-responsive element binding protein (DREB), ethylene response factor (ERF), pearl millet, transcriptionalanalysis

## Abstract

The apetala2/ethylene response factor (AP2/ERF) gene family plays a crucial role in regulating plant growth and development and responding to different abiotic stresses (e.g., drought, heat, cold, and salinity). However, the knowledge of the ERF family in pearl millet remains limited. Here, a total of 167 high-confidence *PgERF* genes are identified and divided into five subgroups based on gene-conserved structure and phylogenetic analysis. Forty-one pairs of segmental duplication are found using collinear analysis. Nucleotide substitution analysis reveals these duplicated pairs are under positive purification, indicating they are actively responding to natural selection. Comprehensive transcriptomic analysis reveals that *PgERF* genesare preferentially expressed in the imbibed seeds and stem (tilling stage) and respond to heat, drought, and salt stress. Prediction of the *cis*-regulatory element by the PlantCARE program indicates that *PgERF* genes are involved in responses to environmental stimuli. Using reverse transcription quantitative real-time PCR (RT-qPCR), expression profiles of eleven selected *PgERF* genes are monitored in various tissues and during different abiotic stresses. Transcript levels of each *PgERF* gene exhibit significant changes during stress treatments. Notably, the *PgERF7* gene is the only candidate that can be induced by all adverse conditions. Furthermore, four *PgERF* genes (i.e., *PgERF22*, *PgERF37*, *PgERF88*, and *PgERF155*) are shown to be involved in the ABA-dependent signaling pathway. These results provide useful bioinformatic and transcriptional information for understanding the roles of the pearl millet *ERF* gene family in adaptation to climate change.

## 1. Introduction

Deleterious environmental conditions, such as soil water limitations, salinity, and extreme ambient temperatures, can severely affect many biological processes in plants. To cope with these abiotic stresses, plants have evolved sophisticated response mechanisms [1,2,3,4]. Transcription factors (TFs) are a class of important functional proteins that govern the expressions of genes through binding to the *cis*-regulatory elements (CREs) in their promoters [5].

The apetala2/ethylene response factor (AP2/ERF) TF super-family is present widely in eukaryotes (Table S1) and is known to regulate plant development and responses to abiotic stresses [6,7,8]. The typical AP2/ERF protein contains one or two AP2 structural domains (about 60 amino acids per domain) to form a three-dimensional structure with three β-folds and one α-helix, and one B3 structural domain [9]. Based on the characteristics of the structural domain, the AP2/ERF TFs are categorized into five subfamilies (e.g., AP2, ERF, DREB, RAV, and Soloist) [8]. The well-studied Dehydration-Responsive Element Binding protein (DREB) TFs have been shown to bind to Dehydration-Responsive Elements (DREs) to activate the expressions of downstream genes during responses to various abiotic stresses. Overexpression of DREB subfamily genes in plants hasbeen found to significantly enhance plant tolerance to abiotic stresses. For instance, overexpression of the *OsDREB1A* or *OsDREB2A* gene in rice plants improves drought and salt stress tolerance [10,11]. Transgenic rice and Arabidopsis plants overexpressing the *OsDREB1F* gene show enhanced tolerance to salt, drought, and low-temperature stresses [12]. *TaDREB3* and *ZmDREB2A* have been shown to increase tolerance to drought stress [13,14]. The TFs in the ERF, AP2, or RAV subfamily also function as master regulators to regulate host tolerance to various environmental stimuli. For example, overexpression of *AtERF75* (*RAP2.2*) gene in Arabidopsis improves resistance to gray mold infection [15]. The *OsERF109* gene is a member of the ERF subfamily and has been shown to negatively regulate drought tolerance and ethylene biosynthesis [16]. Overexpression of the *TaERF3* gene in wheat significantly increases tolerance to salt and drought stresses [17]. In addition, overexpression of *AtRAV1/2* in Arabidopsis enhances tolerance to drought stress [18].

Pearl millet (*Pennisetum glaucum* (L.) R. Br.) is a C4 grass belonging to the *Panicoideae* subfamily and has superior vitality under various environmental conditions. Moreover, pearl millet has higher photosynthetic efficiency and biomass compared to other cereal crops, enabling it to be a crop of high economic value that is suitable to be grown in less developed and resource-limited countries. Recently, the genome of pearl millet has been fully sequenced and annotated. Consequently, pearl millet is now considered an ideal model for the studies of stress tolerance in many cereal crops [19,20,21,22,23,24,25]. Despite several pearl millet TF gene families (i.e., WRKY, NAC, and MYB) having been characterized [26,27,28,29], our understanding of the functions of AP2/ERF family genes in response to abiotic stress remains limited. In this study, we aim to identify and characterize the full pearl millet AP2/ERF family members. Given that AP2/ERF TFs are extensively involved in crop abiotic stress response, we are also interested in investigating their transcriptional changes during different stress treatments in order to provide clues for further gene functional characterization.

## 2. Results

### 2.1. Genome-Wide Identification and Phylogenetic Analysis of PgERF Family Genes

**Figure 1:**
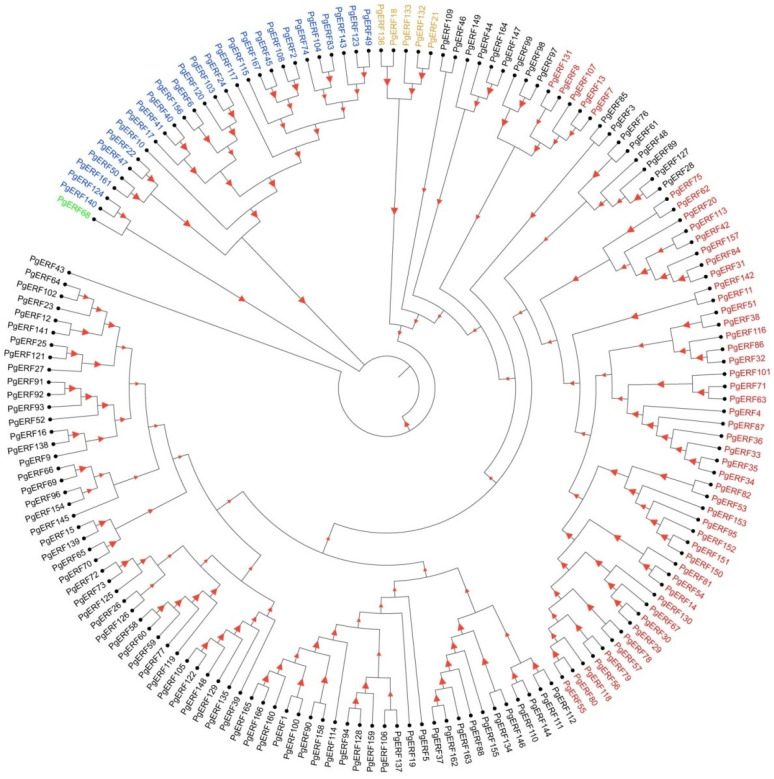
Phylogenetic analysis of *PgERF* gene family. Phylogenetic tree was constructed using MEGA11 with 1000 bootstrap replications based on a full-length amino acid sequence alignment of 167 high-fidelity PgERF proteins. The higher the bootstrap value for a particular branch, the larger the size of the red triangle. Members belonging to ERF, DREB, AP2, RAV, and Soloist subfamilies are represented in black, red, blue, orange, and green fonts, respectively.

**Figure 2:**
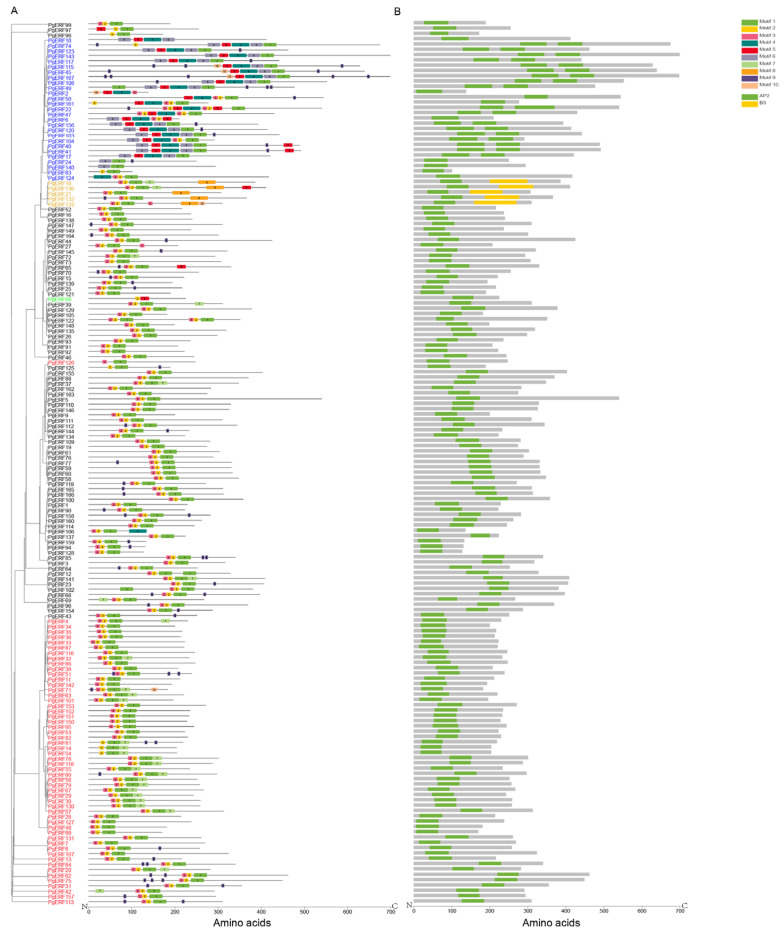
Distributions of conserved motifs (**A**) and domains (**B**) of PgERF proteins. Members belonging to ERF, DREB, AP2, RAV, and Soloist subfamilies are represented in black, red, blue, orange, and green fonts, respectively. Conserved motifs and domains are, respectively, predicted by MEME Suite 5.4.1 and visualized using TBtool v2.052.

A total of 167 *PgERF* family genes were retrieved from the public database and are listed in Table S2. These *PgERF* candidate genes were found on sevenchromosomes. Based on their chromosol locations, they were designated as *PgERF1* to *PgERF167* (Figure 1). The structures of their conserved domains (AP2 and B3) were found to be similar to those of other plant species (Figure 2). According to the distribution patterns of these structural domains, the PgERF family proteins were classified into five subgroups: 27 proteins in the AP2 subgroup, 51 proteins in the DREB subgroup, 83 proteins in the ERF subgroup, 5 proteins in the RAV subgroup, and 1 protein in the Soloist subgroup (Figure 1). The result of subcellular localization prediction showed that 69% of PgERF proteins (116/167) were localized in the nucleus (Table S2).

### 2.2. Conserved Motif and Gene Structural Analysis of PgERF Genes

Proteins in the same group are considered to have similar conserved motifs [30]. In this study, a total of ten motifs were predicted in the PgERF proteins using the MEME software. These predicted motifs were named Motif 1 to Motif 10 (Figure 2A). Motifs 1, 2, and 3 were the most common motifs and were found in the ERF, DREB, AP2, and RAV subgroup proteins. Motifs 2 and 5 were only found in the Soloist subgroup protein, Motif 7 was found to be specific to the ERF and DREB subgroup proteins, and Motif 8 was only found in the RAV subgroup proteins. Other motifs were found to be distributedacross the five subgroups. The transcriptional repressor EAR motif, which contains two signature sequences (DLNx(x)P and LxLxL), was originally found in the members of the Arabidopsis ERF family and has been shown to play important roles in plant development, stress response, and hormone signaling [31,32]. In this study, the EAR motif was found in 34 PgERF proteins, 22 PgERF proteins contained the LxLxL signature sequence, 16 PgERF proteins contained the DLNx(x)P signature sequence, and 4 PgERF proteins contained both signature sequences (Table S2). Five RAV subgroup proteins had both AP2 and B3 domains, whereas other members had only the AP2 domain (Figure 2B). Furthermore, the members of the AP2 subgroup had more exons than thoseof the members of other subgroups (Table S2, Figure S2).

### 2.3. Syntenic Analysis of PgERF Family Genes

**Figure 3:**
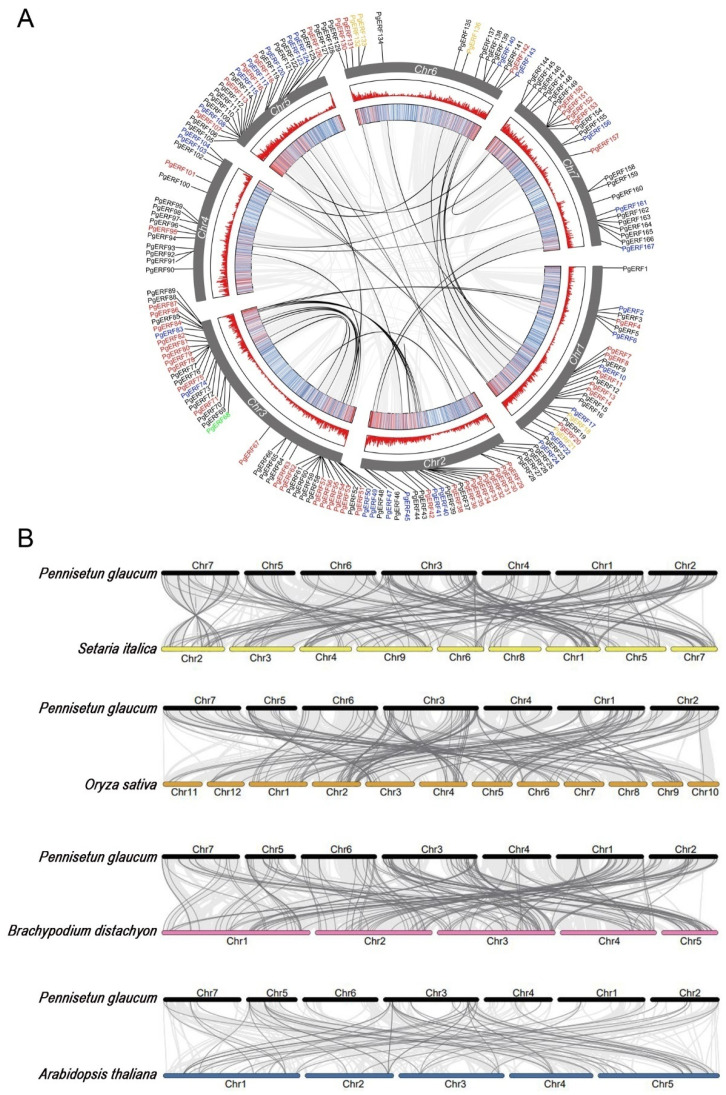
Chromosomal localizations, synteny, and collinearity analysis of *PgERF* gene family. (**A**) Circos plot of *ERF* genes in *P. glaucum* genome. Members belonging to ERF, DREB, AP2, RAV, and Soloist subfamilies are represented in black, red, blue, orange, and green fonts, respectively. The syntenic *PgERF* gene pairs are indicated in black lines. (**B**) Colinearity plot of *ERF* genes between pearl millet and other model species. The light gray lines in the background indicate the collinear region within pearl millet and foxtail millet (pink), rice (green), *Brachypodium* (orange), and Arabidopsis (blue) genomes, while dark gray lines indicate syntenic *ERF* gene pairs, respectively.

Subsequent within-species covariance analysis was performed to explore homologous genes and their evolutionary relationships. As shown in Figure 3, a total of 41 collinear pairs were detected, including 6 pairs of AP2s, 19 pairs of DREBs, 15 pairs of ERFs, and 1 pair of RAV. Gene duplication is known as the main cause of new gene creation and plays a vital role in the evolution and expansion of plant gene families [33]. In this study, all the collinear pairs were formed through segmental duplications but not the tandem duplication (Table S3). The rate of non-synonymous/synonymous (Ka/Ks) was then used to determine the selective pressures during genome evolution. The Ka/Ks ratios for the 41 duplicated gene pairs were all below 1. This finding suggests that the *PgERF* family gene undergoes purifying selection after the gene duplication events.

Next, we investigated the direct homology between the *PgERF* genes and that of foxtail millet, *Brachypodium*, rice, and Arabidopsis. A total of 188, 175, 169, and 58 ortholog *ERF* gene pairs were identified between the pearl millet and other analyzed plant species, respectively (Table S4). As expected, pearl millet had the most *ERF* ortholog pairs with fox millet because of their closest kinship. The result of Ka/Ks analysis of orthologous *ERF* gene pairs between pearl millet and the other four species revealed that the rates of synonymous substitutions in pearl millet were only slightly lower than thoseof foxtail millet and *Brachypodium*.

### 2.4. Identification of Cis-Regulatory Elements (CREs) in PgERF Promoters

Analyses of the distributions of abiotic stress-associated CREs in the promoter regions of *PgERF* genes showed that the ABA-responsive elements (ABREs) and the drought-responsive elements (DREs) were the most abundant phytohormone and environmental stimulus-related CREs (Figure S1 and Table S5). These ABREs and DREs were found in the promoters of 150 and 96 *PgERF* genes, respectively. Notably, *PgERF34* and *PgERF41* genes had the highest (38) and the lowest (3) CRE numbers, respectively. No CRE was found in the promoters of *PgERF26*, *PgERF27*, *PgERF28*, and *PgERF130* genes.

### 2.5. Transcriptional Analyses of PgERF Genes

**Figure 4:**
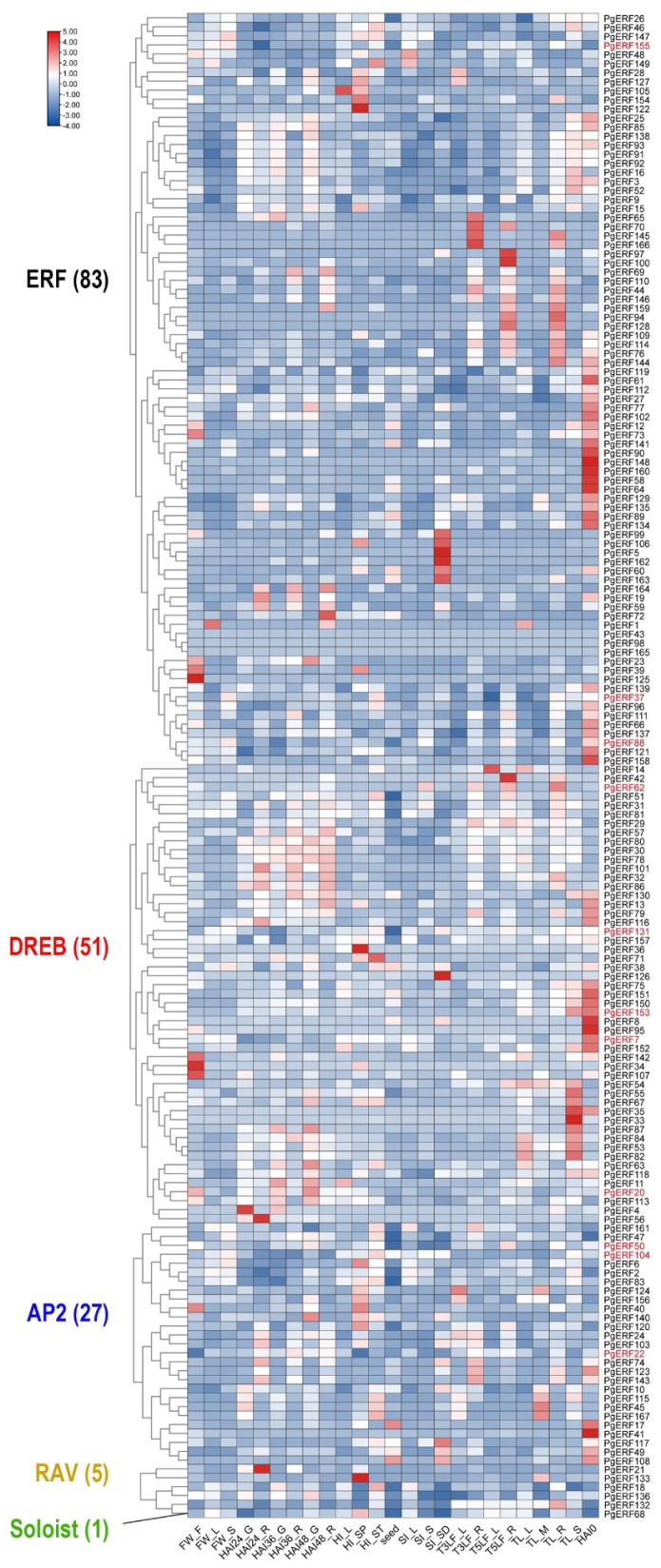
Heat map of the tissue-specific expression profiles of the pearl millet ERF family genes. Transcriptome data wereextracted from public database (Milletdb, http://milletdb.novogene.com, accessed on 15 August 2023). FW_F, flowering stage spike; FW_F, flowering stage leaf; FW_S, flowering stage stem; HAI24_G, imbibition after 24 h germ; HAI24_R, imbibition after 24 h radicle; HAI36_G, imbibition after 36 h germ; HAI36_R, imbibition after 36 h radicle; HAI48_G, imbibition after 48 h germ; HAI48_R, imbibition after 48 h radicle; HI_L, heading stage leaf; HI_SP, heading stage spike; HI_ST, heading stage stem; Seed, ripening seed; SI_L, dough stage leaf; SI_S, dough stage stem; SI_SD, dough stage spike; T3LF_L, three leaf stage leaf; T3LF_R, three leaf stage root; T5LF_L, five leaf stage leaf; T5LF_R, five leaf stage root; TL_L, tillering stage tiller tissue; TL_R, tillering stage root; TL_S, tillering stage stem; HAI0, imbibition seed. The font color of subgroup is as shown in Figure 2. Eleven *PgERF* genes examined by RT-qPCR are shown in red font. The blue–white–red tricolor bar beside the heat map represents their relative fold changes.

**Figure 5:**
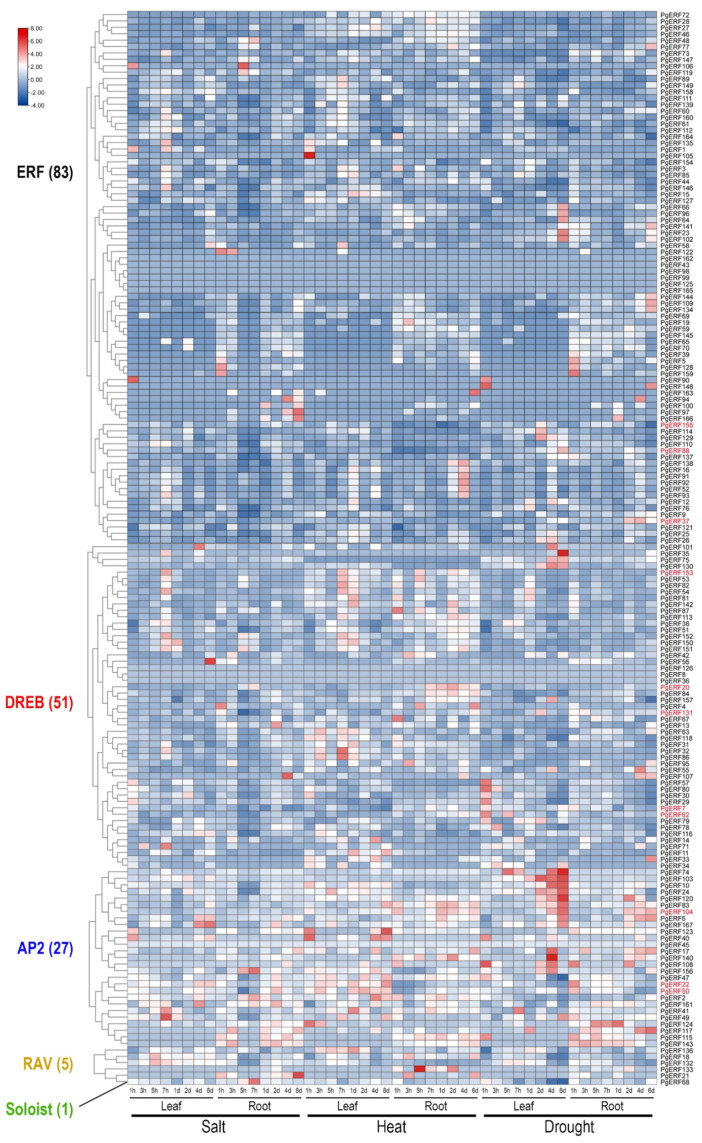
Heat map of the time-course expression profiles of the pearl millet ERF family genes in response to salt, heat, and drought stress. Transcriptome data wereextracted from public database (Milletdb, http://milletdb.novogene.com, accessed on 15 August 2023). The font color of subgroup is as shown in Figure 2. Eleven *PgERF* genes examined by RT-qPCR are shown in red font. The blue–white–red tricolor bar beside the heat map represents their relative fold changes.

To provide an overview of the expression patterns of *PgERF* genes, we extracted the relevant transcriptome data from a public data repository as described in [34]. Of the 167 *PgERF* genes, 57 were preferentially expressed in the imbibed seeds, and 30 were preferentially expressed in the stems during the tilling stage (Figure 4 and Table S6). Under various abiotic stresses, the *PgERF* genes in the AP2 subgroup exhibited significant changes in both leaves and roots (Figure 5 and Table S7). Under the heat and drought stress, but not the salt stress, the *PgDREB* genes showed greater transcriptional changes in the leaves. 

Based on the transcriptome results and literature mining, we subsequently selected eleven *PgERF* genes, including fiveDREB subgroup genes (i.e., *PgERF7*, *PgERF20*, *PgERF62*, *PgERF131*, and *PgERF153*), threeERF subgroup genes (i.e., *PgERF37*, *PgERF88*, and *PgERF155*), and threeAP2 subgroup genes (i.e., *PgERF22*, *PgERF50*, and *PgERF104*), and analyzed their expressions in different tissues, at different growth stages, and under different abiotic stresses through RT-qPCR. The orthologs of these selected genes in other cereal crops have been demonstrated to be involved in response to abiotic stresses.

**Figure 6:**
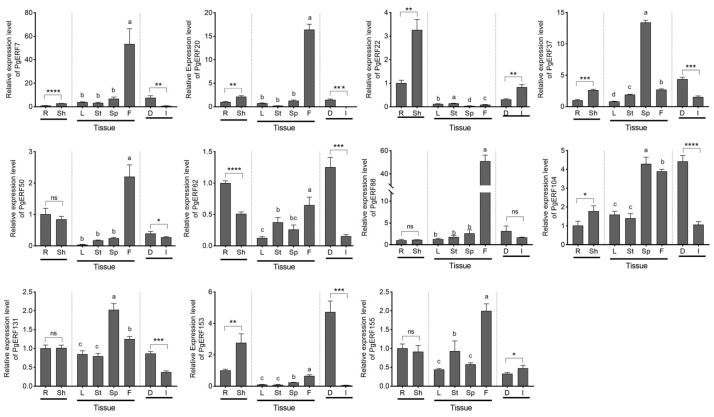
Transcriptional analysis of eleven *PgERF* genes in different organs. Transcript level of each *PgERF* gene in root is set as control. Sh and R, shoot and root of three-week-old seedling; L, mature leaves; St, stems; Sp, spikes; F, flowers; D, dry seeds; I, germinating seeds. The data represent the mean values of three biological replicates ± SD. Statistical significance of differences was tested by one-way ANOVA and Tukey’s analysis (*p* < 0.01), indicated by lowercase letters, or by student’s *t*-test (ns, not significant; * *p* < 0.05; ** *p* < 0.01; *** *p* < 0.001; **** *p* < 0.0001).

The results showed that during the vegetative growth stage, the transcript levels of *PgERF7*, *PgERF20*, *PgERF22*, *PgERF37*, *PgERF104*, and *PgERF153* genes in the shoots were significantly higher than that in the roots, whereas the transcript level of *PgERF62* gene was much higher in the roots (Figure 6). During the reproductive stage, the *PgERF* genes were preferentially expressed in flowers and spikes. Notably, the expression levels of eight *PgERF* genes in dry seeds were significantly higher than thosein germinating seeds; only two *PgERF* genes (i.e., *PgERF22* and *PgERF155*) had higher expression levels in germinating seeds.

**Figure 7:**
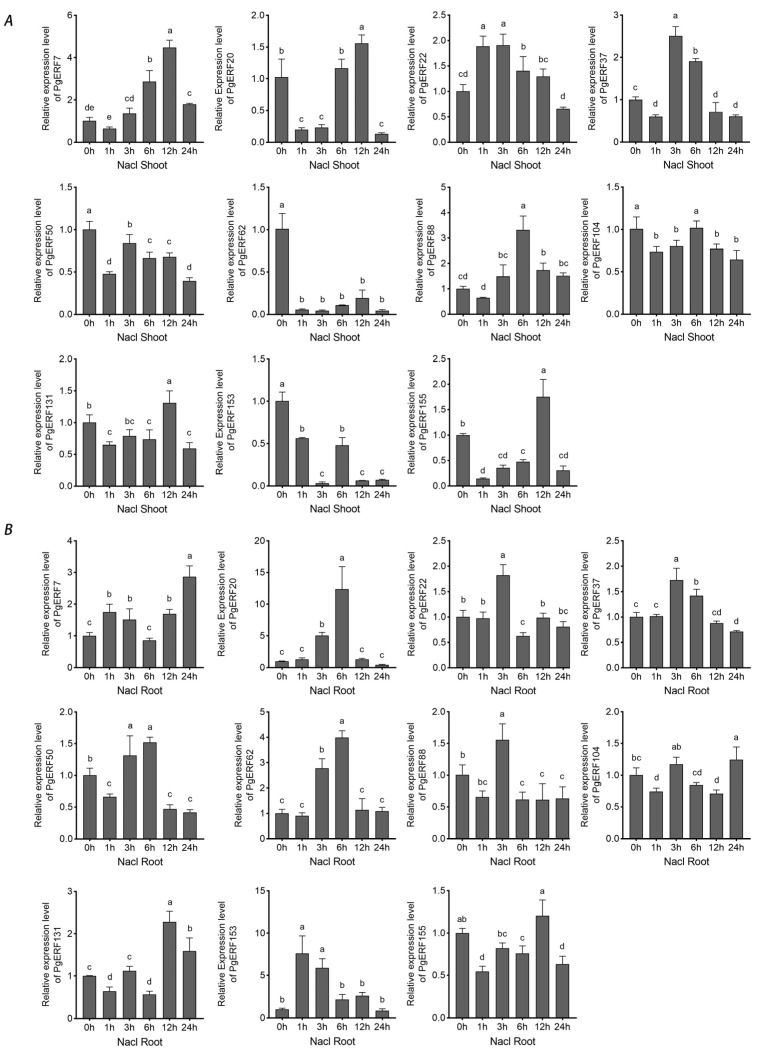
Transcriptional analysis of eleven *PgERF* genes in response to NaCl treatment (150 mM NaCl). Shoot (**A**) and root (**B**) tissues are separately collected from three-week-old hydroponically grown seedlings at the indicated time points. The transcript level of each gene is quantified by using RT-qPCR, and its expression level at 0 h is set as control. The data represent the mean values of three biological replicates ± SD. Statistical significance of differences was tested by one-way ANOVA and Tukey’s analysis (*p* < 0.01) and is indicated by lowercase letters.

**Figure 8:**
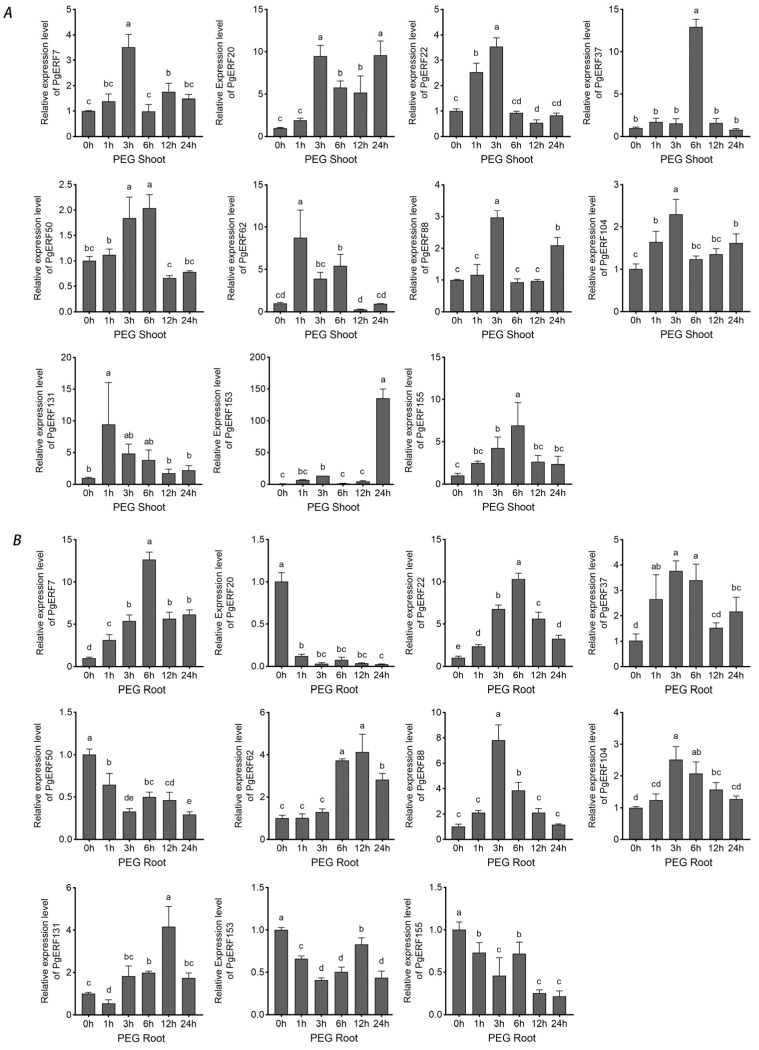
Transcriptional analysis of eleven *PgERF* genes in response to PEG treatment (20% PEG6000, *w*/*v*). Shoot (**A**) and root (**B**) tissues are separately collected from three-week-old hydroponically grown seedlings at the indicated time points. The transcript level of each gene is quantified by using RT-qPCR, and its expression level at 0 h is set as control. The data represent the mean values of three biological replicates ± SD. Statistical significance of differences was tested by one-way ANOVA and Tukey’s analysis (*p* < 0.01) and is indicated by lowercase letters.

**Figure 9:**
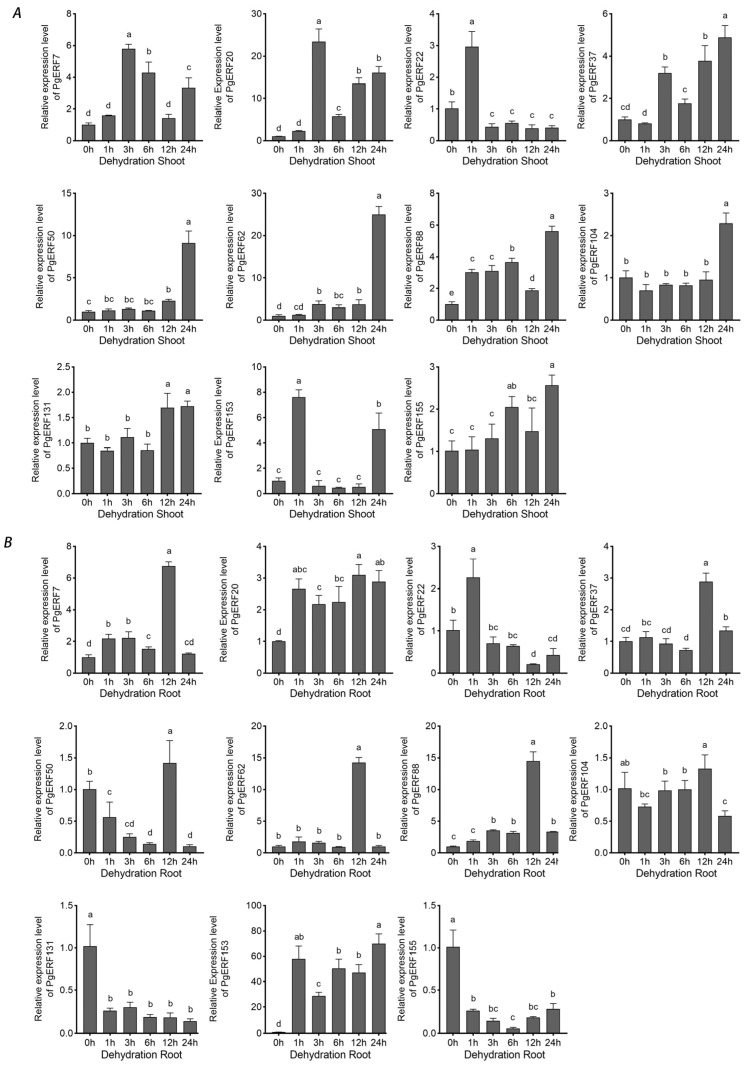
Transcriptional analysis of eleven *PgERF* genes during dehydration treatment. Shoot (**A**) and root (**B**) tissues are separately collected from three-week-old dehydrated seedlings at the indicated time points. The transcript level of each gene is quantified by using RT-qPCR, and its expression level at 0 h is set as control. The data represent the mean values of three biological replicates ± SD. Statistical significance of differences was tested by one-way ANOVA and Tukey’s analysis (*p* < 0.01) and is indicated by lowercase letters.

During osmotic (i.e., NaCl and PEG) treatment and dehydration, eleven *PgERF* genes exhibited different expression patterns in shoot and root tissues (Figure 7, Figure 8 and Figure 9). Under dehydration and PEG treatments, all genes exhibited induction in the shoot, which was different from that under NaCl treatment. On the other hand, the significant down-regulation of these genes wasonly observed in dehydrated or PEG-treated root tissues. For instance, *PgERF20*, *PgERF131*, and *PgERF155* genes in the root were substantially down-regulated after 1 h of PEG or dehydration treatment. Similar trends of transcriptional changes were found in the *PgERF22* gene during dehydration and in the *PgERF37* gene during NaCl treatment. Notably, only the *PgERF7* gene was strongly induced by all three treatments in both shoot and root tissues.

**Figure 10:**
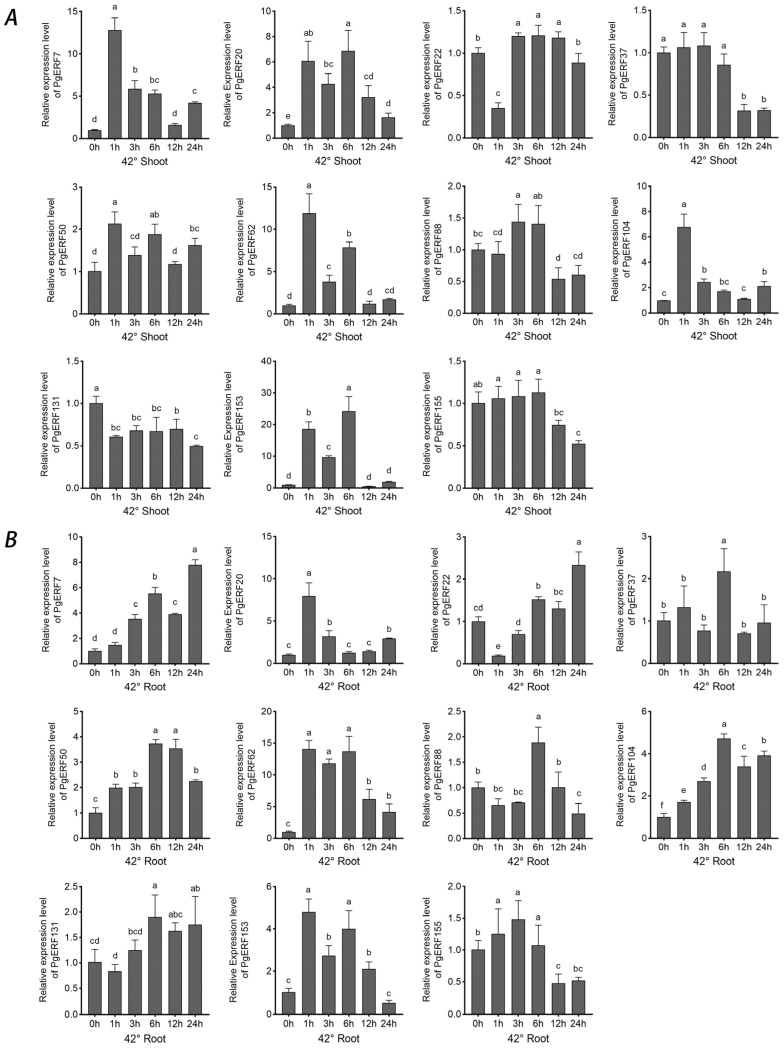
Transcriptional analysis of eleven *PgERF* genes in response to heat stress (42 °C). Shoot (**A**) and root (**B**) tissues are separately collected from three-week-old stressed seedlings at the indicated time points. The transcript level of each gene is quantified by using RT-qPCR, and its expression level at 0 h is set as control. The data represent the mean values of three biological replicates ± SD. Statistical significance of differences was tested by one-way ANOVA and Tukey’s analysis (*p* < 0.01) and is indicated by lowercase letters.

**Figure 11:**
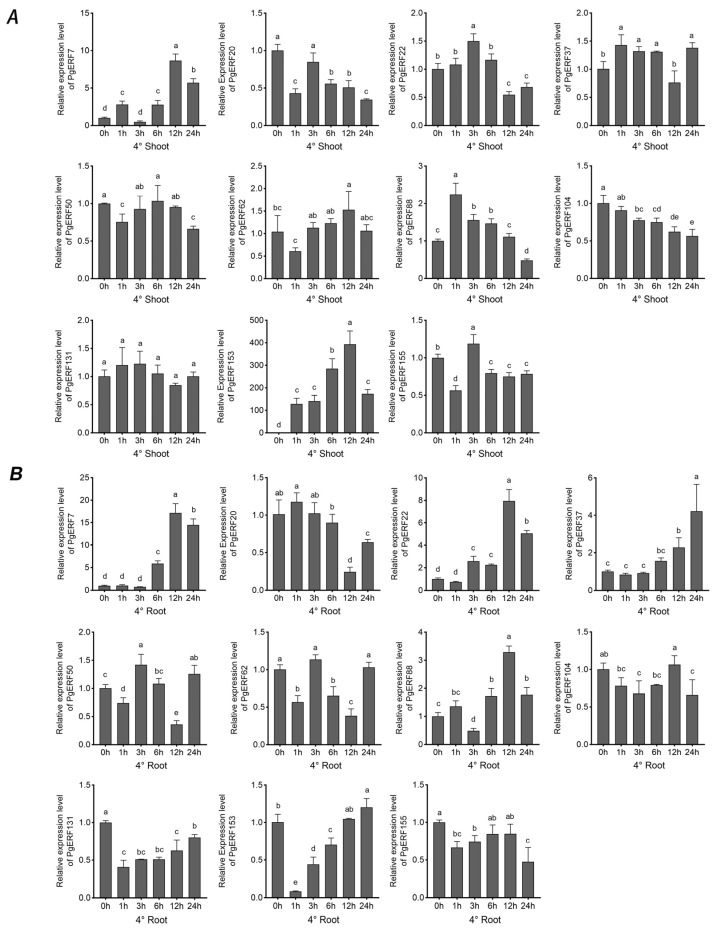
Transcriptional analysis of eleven *PgERF* genes in response to low temperature (4 °C). Shoot (**A**) and root (**B**) tissues are separately collected from three-week-old stressed seedlings at the indicated time points. The transcript level of each gene is quantified by using RT-qPCR, and its expression level at 0 h is set as control. The data represent the mean values of three biological replicates ± SD. Statistical significance of differences was tested by one-way ANOVA and Tukey’s analysis (*p* < 0.01) and is indicated by lowercase letters.

These 11 selected *PgERF* genes also exhibited differential expressions in response to heat (42 °C) and cold (4 °C) stress (Figure 10 and Figure 11). In general, the heat stress-induced transcriptional changes were more pronounced in the shoots than in the roots. On the other hand, fluctuating expression patterns of *PgERF20*, *PgERF50*, and *PgERF62* genes were detected in the shoots and roots during cold (4 °C) stress. Unsurprisingly, the transcript level of the *PgERF7* gene was significantly up-regulated in the shoots and roots during both heat and cold stresses. The strongest transcriptional induction (>100-fold changes) was observed for the *PgERF153* gene in the cold-stressed shoots.

**Figure 12:**
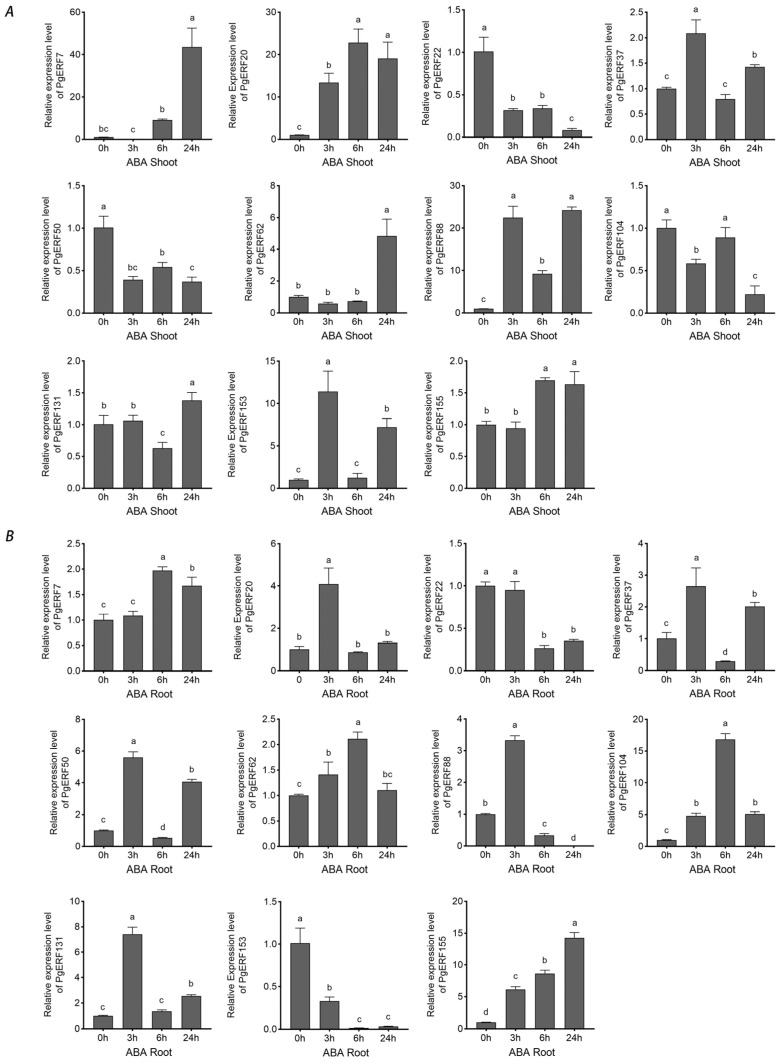
Transcriptional analysis of eleven *PgERF* genes in response to ABA treatment (100 μM). Shoot (**A**) and root (**B**) tissues are separately collected from three-week-old stressed seedlings at the indicated time points. The transcript level of each gene is quantified by using RT-qPCR, and its expression level at 0 h is set as control. The data represent the mean values of three biological replicates ± SD. Statistical significance of differences was tested by one-way ANOVA and Tukey’s analysis (*p* < 0.01), and is indicated by lowercase letters.

ABA is one of the most well-studied phytohormones that mediates signaling transduction during abiotic stresses [35]. Therefore, we investigated the expressions of these 11 *PgERF* genes in the ABA-treated plants (Figure 12). Our results showed that under ABA treatment, the expressions of *PgERF7*, *PgERF20*, *PgERF62*, and *PgERF155* genes were up-regulated, and the expression of *PgERF22* gene was down-regulated in both shoots and roots. In addition, the expressions of *PgERF37*, *PgERF50*, *PgERF88*, and *PgERF131* genes fluctuated, similar to that in the shoots and roots of the plants under other stress treatments. In summary, our results indicate that *PgERF* genes significantly respond to different abiotic stresses in a stress- and tissue-specific manner.

## 3. Discussion

The AP2/ERF gene family is one of the largest gene families in plants and encodes many transcription factors to actively regulate numerous biological processes [36]. To date, comprehensive analyses of pearl millet AP2/ERF family genes are lacking. In this study, we identify 167 high-confidence and intact AP2/ERF family genes in the pearl millet public genome database (Table S2). These genes account for 0.47% of all pearl millet genes and share high sequence identities with that found in rice, *Brachypodium,* and fox millet. Eukaryotes undergo frequent genome polyploidization through tandem, segmental, and/or genome-wide duplication events [37]. Through a genome-wide survey, we have discovered 41 segmental duplicated pairs of *PgERF* genes (Figure 3 and Table S3). Because tandem duplications are more common for the ERF family genes in other plant species [38,39,40], we speculate that the *PgERF* genes are under positive purification in response to natural selection.

The AP2/ERF TFs, especially the TFs in the DREB subgroup, play crucial roles in the regulations of plant growth, development, responses to abiotic and biotic stresses, and signaling pathways. The DREB TFs have been shown to recognize DRE/CRT *cis*-elements to confer resistance to abiotic stresses [41]. Overexpression of DREB subgroup genes in plants can significantly improve abiotic stress tolerance [13,42,43,44]. The *PgDREB2A* (*PgERF7* in this study) gene has been reported to be induced by cold and drought stress [45]. Here, we find that the expression of the *PgERF7* gene is highly induced in the shoots and roots of the plants under all the tested treatments (Figure 7, Figure 8, Figure 9, Figure 10, Figure 11 and Figure 12), which is consistent with previous results. Ectopic expressions of the *PgERF7* gene in Arabidopsis and tobacco plants enhance their tolerance to abiotic stresses [46,47]. In order to identify the downstream genes of *PgERF7*, we searched the co-expressed genes of *PgERF7* and the other four candidate *PgDREB* genes under salt, heat, or drought stress (Table S8). Because abiotic stresses often occur simultaneously, we focus our studies on the co-expressed genes found in plants under more than one stress. Our results show that five genes (*PMA5G06042.1*, *PMA6G00641.1*, *PMA1G06009.1*, *PMA5G05409.1*, and *PMA2G04696.1*) are co-expressed with *PgERF7* gene under the heat and salt stresses. The *PMA4G02105.1* gene is co-expressed under salt and drought stresses, while the *PMA2G04302.1* gene is co-expressed under drought and heat stresses. Because several DREs are found in the promoters of these co-expressed genes, except *PMA1G00309.1* and *PMA2G04302.1*, we speculate that the stress-inducible *PgERF7* gene can directly trigger their expressions. For *PgDREB* family genes, we find that the *PgERF62* gene is co-expressed with two MYB TF encoding genes (*PMA2G00728.1* and *PMA6G03954.1*) under drought and heat stresses. Previously, we reported that *PMA2G00728.1* and *PgMYB159* genes are involved in the response to abiotic stress and ABA treatment [28]. Thus, we consider that the *PgERF62* gene is likely an upstream gene to regulate the signaling transduction cascade. The *PgERF153* gene belongs to the DREB subgroup and responds strongly to drought and temperature stresses (Figure 9, Figure 10 and Figure 11). Its homologs in rice, wheat, maize, and soybean have been demonstrated to reinforce tolerance to abiotic stresses [11,48,49,50,51], making *PgERF153* an interesting gene for further functional characterization.

*PgERF* genes outside the DREB subgroup are also involved in abiotic stresses. For example, the segmentally duplicated pair (*PgERF37* and *PgERF88*) genes are both induced by dehydration or osmotic treatment (Figure 7, Figure 8 and Figure 9). Their expression patterns are, however, not identical, due likely to the differences between their CREs. Their orthologs in rice, the *OsERF71* gene, havebeen shown to enhance tolerance to drought stress and increase grain yield [52]. Overexpression of *AtERF74* gene in Arabidopsis affects the homeostasis of hydrogen peroxide (H_2_O_2_), resulting in an enhanced stress tolerance [53]. Whether *PgERF37* and *PgERF88* genes play similar functions in pearl millet deserves further investigation.

ABA is an important long-distance trafficking messenger responsible for the perceptions of environmental stimuli and activation of signaling transduction [35]. All tested *PgERF* genes positively respond to ABA treatment, and some of them exhibit similar expression patterns in the assayed shoots and roots (Figure 12). Furthermore, our results show that several *PgERF* genes respond similarly to the ABA treatment and other abiotic stresses. For instance, the dehydration- and ABA treatment-induced transcriptional changes of *PgERF22*, *PgERF37*, and *PgERF155* genes in shoots are comparable. Similar transcriptional changes are also observed for the *PgERF88* gene in both shoots and roots during the PEG treatment. Considering the ubiquity of ABRE in the promoter region, we consider that these *PgERF* genes are likely involved in the ABA-dependent signaling pathway in response to drought stress. 

Lately, multiple novel drought stress-responsive microRNAs (miRNA) have beenidentified from the genomic resources of pearl millet, sorghum, foxtail millet, finger millet, and proso millet through in silico approaches [54]. By searching their database, 23 pearl millet miRNAs are predicted to target 67 different *PgERF* genes (Table S9), suggesting they might be involved in drought-inducible post-transcriptional regulation.

## 4. Materials and Methods

### 4.1. Identification and Phylogenetic Analysis of Pearl Millet AP2/ERF Family Genes

The whole genomic data of pearl millet cv. PI537069 was acquired from of public genome sequencing database (Milletdb, http://milletdb.novogene.com, accessed on 30 July 2023) [34]. The Hidden Markov Model (HMM) profile of AP2/ERF conserved domains (PF00847) [55] was downloaded from the Pfam database v35.0 [56] and employed to identify the pearl millet AP2/ERF (PgERF) protein sequences using the HMMER 3.0 program [57]. The output proteins with an e-value ≤ 1 × 10^−5^ were collected and considered as the candidate members of the PgERF family proteins. The NCBI-CDD web server [58] and the SMART program [59] were then used to further confirm the structures of PgERF proteins (Files S1 and S2). Physicochemical properties of the PgERF proteins were predicted using the online ExPASy software (https://www.expasy.org/tools/protparam.html, accessed on 30 July 2023). A neighbor-joining (NJ) phylogenetic tree was then constructed and visualized using the MEGA11 [60] and the iTOL v6 software [61], respectively. Analysis of bootstrap with 1000 replicates was performed to calculate the reliability of the NJ tree. The subcellular localizations of the proteins were conducted using a tool in the PSORT website (https://wolfpsort.hgc.jp/, accessed on 30 July 2023).

### 4.2. Synteny Analysis of PgERF Family Genes

Comparison of sequence homology among PgERF proteins and those of other plant species [e.g., foxtail millet (*Setaria italica*), rice, *Brachypodium distachyon*, and *Arabidopsis thaliana*], genomic sequences of these plant species were obtained from the Ensemble Plants Database (http://plants.ensembl.org/index.html, accessed on 30 July 2023). Covariance among these analyzed sequences was calculated using the MCScanX software [62] and visualized using the Advanced circle function of TBtool v2.052 [63]. To further estimate the replication events of *PgERF* family genes, the Ka (non-synonymous substitutions) and the Ks (synonymous substitutions) values of the cognate gene pairs were calculated using the Ka/Ks calculation function of DnaSP6 [64].

### 4.3. Analysis of Conserved Motifs and Gene Structures

The conserved motifs in the PgERF family proteins were predicted using the MEME Suite 5.5.3 website tool [65]. The maximum conserved motif number found in this study was ten. Other parameters were used as the default values. The information of exons and introns in the *PgERF* family genes were retrieved from the pearl millet genomic information data.

### 4.4. Analysis of PgERF Gene Promoters

The upstream genomic DNA sequences (2.0 kb above the start codon) of the *PgERF* family (File S3) genes were used in the promoter analysis. The stress related *cis*-regulatory elements were predicted using the PlantCARE program (http://bioinformatics.psb.ugent.be/webtools/plantcare/html/, accessed on 30 July 2023) [66].

### 4.5. Transcriptome Analysis of PgERF Family Genes

For overview of the tissue-specific and stress-inducible expression changes of *PgERF* genes, transcript data were retrieved from the Pearl millet public database (Milletdb, http://milletdb.novogene.com, accessed on 15 August 2023). The expression profiles of all *PgERF* genes were exhibited as Transcripts Per Kilobase of exon model per Million mapped reads (TPM) values and plotted by dividing the values obtained from the treatment group by the blank group, and the transformed logs were presented in TBtool v2.052.

### 4.6. Quantitative RT-PCR (RT-qPCR) Analysis

Pearl millet plant growth, stress treatments, RNA extraction, and cDNA synthesis were performed as described previously [28]. Briefly, 3-week-old plantlets with uniform and robust growth were selected for abiotic stress treatments. For osmotic stress, the seedlings were placed in a nutrient solution containing 20% (*w*/*v*) PEG6000 (KeMing Technology Co., Ltd., Zhangjiang, China) and 150 mM NaCl (KeMing Technology Co., Ltd., Zhangjiang, China), respectively. For dehydration treatment, the seedlings were placed on the Whatman 3MM paper to dry in ambient temperature. For temperature stress, the seedlings were transferred to growth chamber at 42 °C and 4 °C, respectively, to simulate heat and cold stresses. For ABA treatment, a solution containing 100 μM ABA (KeMing Technology Co., Ltd., Zhangjiang, China) was applied to the seedlings. The shoot and root tissues were respectively collected at indicated time points, quickly frozen in liquid nitrogen, and stored in −80 °C freezer for downstream analysis. For tissue specific expression analysis, the root and shoot tissue of three-week-old hydroponically grown seedlings, leaves, stems, and spikes of soil-grown mature plants, dry and germinating seeds were collected. Expressions of 11 *PgERF* family genes were analyzed through qRT-PCR. The qRT–PCR primers were designed using the Primer Premier 6.25 software (PREMIER Biosoft, San Francisco, CA, USA) and are listed in the Table S10. Quantitative PCR was conducted on a BioRad CFX96 Touch Real-Time PCR Detection System using the TB Green^®^ Fast qPCR Mix (Takara, Shiga, Japan). The expression level of pearl millet EF1α gene (accession: EF694165) was used as the internal control [67]. Each biological sample has three technical replicates and the relative expression of each gene was calculated using the 2^−ΔΔCT^ method as described [68].

### 4.7. Statistical Analysis

All assay data were initially analyzed using an Excel tool and then subjected to statistical analysis using the IBM SPSS Statistics software for one-way ANOVA or student’s *t*-test. The graphic plots were later generated using the GraphPad Prism version 9.3 as instructed (GraphPad Software, Inc., San Diego, CA, USA).

## 5. Conclusions

Pearl millet is a climate-adapted cereal crop capable of growing under various growth conditions. Due to global warming and other environmental changes, we consider the AP2/ERF family genes identified in this study to bea useful gene pool for coping with various abiotic stresses. In the present study, we identify 167 high-confidence pearl millet *AP2/ERF* genes through a genome-wide survey and perform bioinformatics analysis to uncover their physiochemical properties and phylogenetic and collinear relationships. In addition, we analyze the transcriptional levels of eleven *PgERF* genes in different tissues and in response to various abiotic treatments (e.g., NaCl, PEG, dehydration, etc.). The expressions of *PgERF7*, *PgERF37*, *PgERF88*, and *PgERF153* genes are strongly induced by different abiotic stresses and, thus, are worthy offurther functional characterization. These findings not only offer first-hand evidence of *PgERF* genes involved in responding to climate change but also provide new candidates for breeding the next generation of stress-tolerant pearl millet plants through genetic engineering tools.

## Data Availability

The DNA, cDNA, and protein sequences of the pearl millet ERF family in this study can beretrieved from Milletdb (http://milletdb.novogene.com, accessed on 15 July 2023) and are included in Supplementary Materials S1–S3. Plant material is available from the corresponding author upon reasonable request.

## Supplementary Materials

The following supporting information can be downloaded at https://www.mdpi.com/article/10.3390/ijms25052470/s1.

## References

[1] Gupta A., Rico-Medina A., Caño-Delgado A.I. (2020). The physiology of plant responses to drought. Science.

[2] Zhang H., Zhu J., Gong Z., Zhu J.-K. (2022). Abiotic stress responses in plants. Nat. Rev. Genet..

[3] Zhang H., Zhao Y., Zhu J.K. (2020). Thriving under Stress: How Plants Balance Growth and the Stress Response. Dev. Cell.

[4] Takahashi F., Kuromori T., Urano K., Yamaguchi-Shinozaki K., Shinozaki K. (2020). Drought Stress Responses and Resistance in Plants: From Cellular Responses to Long-Distance Intercellular Communication. Front. Plant Sci..

[5] Strader L., Weijers D., Wagner D. (2022). Plant transcription factors—Being in the right place with the right company. Curr. Opin. Plant Biol..

[6] Fukao T., Xu K., Ronald P.C., Bailey-Serres J. (2006). A variable cluster of ethylene response factor-like genes regulates metabolic and developmental acclimation responses to submergence in rice. Plant Cell.

[7] Xu Z.-S., Chen M., Li L.-C., Ma Y.-Z. (2011). Functions and Application of the AP2/ERF Transcription Factor Family in Crop Improvement. J. Integr. Plant Biol..

[8] Mizoi J., Shinozaki K., Yamaguchi-Shinozaki K. (2012). AP2/ERF family transcription factors in plant abiotic stress responses. Biochim. Biophys. Acta.

[9] Kagaya Y., Ohmiya K., Hattori T. (1999). RAV1, a novel DNA-binding protein, binds to bipartite recognition sequence through two distinct DNA-binding domains uniquely found in higher plants. Nucleic Acids Res..

[10] Mallikarjuna G., Mallikarjuna K., Reddy M.K., Kaul T. (2011). Expression of OsDREB2A transcription factor confers enhanced dehydration and salt stress tolerance in rice (*Oryza sativa* L.). Biotechnol. Lett..

[11] Ahmad F., Shah S.H., Jan A. (2023). Overexpression of the DREB1A gene under stress-inducible promoter delays leaf senescence and improves drought tolerance in rice. Cereal Res. Commun..

[12] Wang Q., Guan Y., Wu Y., Chen H., Chen F., Chu C. (2008). Overexpression of a rice OsDREB1F gene increases salt, drought, and low temperature tolerance in both Arabidopsis and rice. Plant Mol. Biol..

[13] Qin F., Kakimoto M., Sakuma Y., Maruyama K., Osakabe Y., Tran L.-S.P., Shinozaki K., Yamaguchi-Shinozaki K. (2007). Regulation and functional analysis of ZmDREB2A in response to drought and heat stresses in *Zea mays* L. Plant J..

[14] Niu X., Luo T., Zhao H., Su Y., Ji W., Li H. (2020). Identification of wheat DREB genes and functional characterization of TaDREB3 in response to abiotic stresses. Gene.

[15] Zhao Y., Wei T., Yin K.-Q., Chen Z., Gu H., Qu L.-J., Qin G. (2012). Arabidopsis RAP2.2 plays an important role in plant resistance to Botrytis cinerea and ethylene responses. New Phytol..

[16] Yu Y., Yang D., Zhou S., Gu J., Wang F., Dong J., Huang R. (2017). The ethylene response factor OsERF109 negatively affects ethylene biosynthesis and drought tolerance in rice. Protoplasma.

[17] Rong W., Qi L., Wang A., Ye X., Du L., Liang H., Xin Z., Zhang Z. (2014). The ERF transcription factor TaERF3 promotes tolerance to salt and drought stresses in wheat. Plant Biotechnol. J..

[18] Matias-Hernandez L., Aguilar-Jaramillo A.E., Marin-Gonzalez E., Suarez-Lopez P., Pelaz S. (2014). RAV genes: Regulation of floral induction and beyond. Ann. Bot..

[19] Sun M., Lin C., Zhang A., Wang X., Yan H., Khan I., Wu B., Feng G., Nie G., Zhang X. (2021). Transcriptome sequencing revealed the molecular mechanism of response of pearl millet root to heat stress. J. Agron. Crop Sci..

[20] Yan H., Sun M., Zhang Z., Jin Y., Zhang A., Lin C., Wu B., He M., Xu B., Wang J. (2023). Pangenomic analysis identifies structural variation associated with heat tolerance in pearl millet. Nat. Genet..

[21] Varshney R.K., Shi C., Thudi M., Mariac C., Wallace J., Qi P., Zhang H., Zhao Y., Wang X., Rathore A. (2017). Pearl millet genome sequence provides a resource to improve agronomic traits in arid environments. Nat. Biotechnol..

[22] Zhang A., Ji Y., Sun M., Lin C., Zhou P., Ren J., Luo D., Wang X., Ma C., Zhang X. (2021). Research on the drought tolerance mechanism of *Pennisetum glaucum* (L.) in the root during the seedling stage. BMC Genom..

[23] Sun M., Huang D., Zhang A., Khan I., Yan H., Wang X., Zhang X., Zhang J., Huang L. (2020). Transcriptome analysis of heat stress and drought stress in pearl millet based on Pacbio full-length transcriptome sequencing. BMC Plant Biol..

[24] Satyavathi C.T., Ambawat S., Khandelwal V., Srivastava R.K. (2021). Pearl Millet: A Climate-Resilient Nutricereal for Mitigating Hidden Hunger and Provide Nutritional Security. Front. Plant Sci..

[25] Ghatak A., Chaturvedi P., Bachmann G., Valledor L., Ramšak Ž., Bazargani M.M., Bajaj P., Jegadeesan S., Li W., Sun X. (2020). Physiological and Proteomic Signatures Reveal Mechanisms of Superior Drought Resilience in Pearl Millet Compared to Wheat. Front. Plant Sci..

[26] Chanwala J., Satpati S., Dixit A., Parida A., Giri M.K., Dey N. (2020). Genome-wide identification and expression analysis of WRKY transcription factors in pearl millet (*Pennisetum glaucum*) under dehydration and salinity stress. BMC Genom..

[27] Dudhate A., Shinde H., Yu P., Tsugama D., Gupta S.K., Liu S., Takano T. (2021). Comprehensive analysis of NAC transcription factor family uncovers drought and salinity stress response in pearl millet (*Pennisetum glaucum*). BMC Genom..

[28] Lin M., Dong Z., Zhou H., Wu G., Xu L., Ying S., Chen M. (2023). Genome-Wide Identification and Transcriptional Analysis of the MYB Gene Family in Pearl Millet (*Pennisetum glaucum*). Int. J. Mol. Sci..

[29] Chanwala J., Khadanga B., Jha D.K., Sandeep I.S., Dey N. (2023). MYB Transcription Factor Family in Pearl Millet: Genome-Wide Identification, Evolutionary Progression and Expression Analysis under Abiotic Stress and Phytohormone Treatments. Plants.

[30] Shoji T., Yuan L. (2021). ERF Gene Clusters: Working together to Regulate Metabolism. Trends Plant Sci..

[31] Ohta M., Matsui K., Hiratsu K., Shinshi H., Ohme-Takagi M. (2001). Repression Domains of Class II ERF Transcriptional Repressors Share an Essential Motif for Active Repression. Plant Cell.

[32] Payankaulam S., Li L.M., Arnosti D.N. (2010). Transcriptional Repression: Conserved and Evolved Features. Curr. Biol..

[33] Cannon S.B., Mitra A., Baumgarten A., Young N.D., May G. (2004). The roles of segmental and tandem gene duplication in the evolution of large gene families in Arabidopsis thaliana. BMC Plant Biol..

[34] Sun M., Yan H., Zhang A., Jin Y., Lin C., Luo L., Wu B., Fan Y., Tian S., Cao X. (2023). Milletdb: A multi-omics database to accelerate the research of functional genomics and molecular breeding of millets. Plant Biotechnol. J..

[35] Waadt R., Seller C.A., Hsu P.-K., Takahashi Y., Munemasa S., Schroeder J.I. (2022). Plant hormone regulation of abiotic stress responses. Nat. Rev. Mol. Cell Biol..

[36] Feng K., Hou X.-L., Xing G.-M., Liu J.-X., Duan A.-Q., Xu Z.-S., Li M.-Y., Zhuang J., Xiong A.-S. (2020). Advances in AP2/ERF super-family transcription factors in plant. Crit. Rev. Biotechnol..

[37] Moore R.C., Purugganan M.D. (2005). The evolutionary dynamics of plant duplicate genes. Curr. Opin. Plant Biol..

[38] Cui L., Feng K., Wang M., Wang M., Deng P., Song W., Nie X. (2016). Genome-wide identification, phylogeny and expression analysis of AP2/ERF transcription factors family in Brachypodium distachyon. BMC Genom..

[39] Nakano T., Suzuki K., Fujimura T., Shinshi H. (2006). Genome-wide analysis of the ERF gene family in Arabidopsis and rice. Plant Physiol..

[40] Lata C., Mishra A.K., Muthamilarasan M., Bonthala V.S., Khan Y., Prasad M. (2014). Genome-wide investigation and expression profiling of AP2/ERF transcription factor superfamily in foxtail millet (*Setaria italica* L.). PLoS ONE.

[41] Lata C., Prasad M. (2011). Role of DREBs in regulation of abiotic stress responses in plants. J. Exp. Bot..

[42] Sakuma Y., Maruyama K., Osakabe Y., Qin F., Seki M., Shinozaki K., Yamaguchi-Shinozaki K. (2006). Functional analysis of an Arabidopsis transcription factor, DREB2A, involved in drought-responsive gene expression. Plant Cell.

[43] Matsukura S., Mizoi J., Yoshida T., Todaka D., Ito Y., Maruyama K., Shinozaki K., Yamaguchi-Shinozaki K. (2010). Comprehensive analysis of rice DREB2-type genes that encode transcription factors involved in the expression of abiotic stress-responsive genes. Mol. Genet. Genom..

[44] Egawa C., Kobayashi F., Ishibashi M., Nakamura T., Nakamura C., Takumi S. (2006). Differential regulation of transcript accumulation and alternative splicing of a DREB2 homolog under abiotic stress conditions in common wheat. Genes Genet. Syst..

[45] Agarwal P., Agarwal P.K., Nair S., Sopory S.K., Reddy M.K. (2007). Stress-inducible DREB2A transcription factor from *Pennisetum glaucum* is a phosphoprotein and its phosphorylation negatively regulates its DNA-binding activity. Mol. Genet. Genom..

[46] Agarwal P., Agarwal P.K., Joshi A.J., Sopory S.K., Reddy M.K. (2009). Overexpression of PgDREB2A transcription factor enhances abiotic stress tolerance and activates downstream stress-responsive genes. Mol. Biol. Rep..

[47] Meena R.P., Ghosh G., Vishwakarma H., Padaria J.C. (2022). Expression of a *Pennisetum glaucum* gene DREB2A confers enhanced heat, drought and salinity tolerance in transgenic Arabidopsis. Mol. Biol. Rep..

[48] Guttikonda S.K., Valliyodan B., Neelakandan A.K., Tran L.-S.P., Kumar R., Quach T.N., Voothuluru P., Gutierrez-Gonzalez J.J., Aldrich D.L., Pallardy S.G. (2014). Overexpression of AtDREB1D transcription factor improves drought tolerance in soybean. Mol. Biol. Rep..

[49] Qin F., Sakuma Y., Li J., Liu Q., Li Y.Q., Shinozaki K., Yamagushi-Shinozaki K.Y. (2004). Cloning and functional analysis of a novel DREB1/CBF transcription factor involved in cold-responsive gene expression in *Zea mays* L. Plant Cell Physiol..

[50] Xu Z.-S., Ni Z.-Y., Liu L., Nie L.-N., Li L.-C., Chen M., Ma Y.-Z. (2008). Characterization of the TaAIDFa gene encoding a CRT/DRE-binding factor responsive to drought, high-salt, and cold stress in wheat. Mol. Genet. Genom..

[51] Ryu J.Y., Hong S.-Y., Jo S.-H., Woo J.-C., Lee S., Park C.-M. (2014). Molecular and functional characterization of cold-responsive C-repeat binding factors from Brachypodium distachyon. BMC Plant Biol..

[52] Lee D.-K., Jung H., Jang G., Jeong J.S., Kim Y.S., Ha S.-H., Do Choi Y., Kim J.-K. (2016). Overexpression of the OsERF71 Transcription Factor Alters Rice Root Structure and Drought Resistance. Plant Physiol..

[53] Yao Y., He R.J., Xie Q.L., Zhao X.H., Deng X.M., He J.B., Song L., He J., Marchant A., Chen X.-Y. (2017). ETHYLENE RESPONSE FACTOR 74 (ERF74) plays an essential role in controlling a respiratory burst oxidase homolog D (RbohD)-dependent mechanism in response to different stresses in Arabidopsis. New Phytol..

[54] Chakraborty A., Viswanath A., Malipatil R., Rathore A., Thirunavukkarasu N. (2020). Structural and Functional Characteristics of miRNAs in Five Strategic Millet Species and Their Utility in Drought Tolerance. Front. Genet..

[55] Mistry J., Chuguransky S., Williams L., Qureshi M., Salazar G.A., Sonnhammer E.L.L., Tosatto S.C.E., Paladin L., Raj S., Richardson L.J. (2021). Pfam: The protein families database in 2021. Nucleic Acids Res..

[56] Paysan-Lafosse T., Blum M., Chuguransky S., Grego T., Pinto B.L., Salazar G.A., Bileschi M.L., Bork P., Bridge A., Colwell L. (2023). InterPro in 2022. Nucleic Acids Res..

[57] Eddy S.R. (2011). Accelerated Profile HMM Searches. PLoS Comput. Biol..

[58] Marchler-Bauer A., Bo Y., Han L., He J., Lanczycki C.J., Lu S., Chitsaz F., Derbyshire M.K., Geer R.C., Gonzales N.R. (2017). CDD/SPARCLE: Functional classification of proteins via subfamily domain architectures. Nucleic Acids Res..

[59] Letunic I., Khedkar S., Bork P. (2021). SMART: Recent updates, new developments and status in 2020. Nucleic Acids Res..

[60] Tamura K., Stecher G., Kumar S. (2021). MEGA11 Molecular Evolutionary Genetics Analysis Version 11. Mol. Biol. Evol..

[61] Letunic I., Bork P. (2019). Interactive Tree Of Life (iTOL) v4: Recent updates and new developments. Nucleic Acids Res..

[62] Wang Y., Tang H., Debarry J.D., Tan X., Li J., Wang X., Lee T.H., Jin H., Marler B., Guo H. (2012). MCScanX: A toolkit for detection and evolutionary analysis of gene synteny and collinearity. Nucleic Acids Res..

[63] Chen C., Chen H., Zhang Y., Thomas H.R., Frank M.H., He Y., Xia R. (2020). TBtools: An Integrative Toolkit Developed for Interactive Analyses of Big Biological Data. Mol. Plant.

[64] Rozas J., Ferrer-Mata A., Carlos Sanchez-DelBarrio J., Guirao-Rico S., Librado P., Ramos-Onsins S.E., Sanchez-Gracia A. (2017). DnaSP 6: DNA Sequence Polymorphism Analysis of Large Data Sets. Mol. Biol. Evol..

[65] Bailey T.L., Boden M., Buske F.A., Frith M., Grant C.E., Clementi L., Ren J., Li W.W., Noble W.S. (2009). MEME SUITE: Tools for motif discovery and searching. Nucleic Acids Res..

[66] Rombauts S., Dehais P., Van Montagu M., Rouze P. (1999). PlantCARE, a plant cis-acting regulatory element database. Nucleic Acids Res..

[67] Reddy P.S., Reddy D.S., Sharma K.K., Bhatnagar-Mathur P., Vadez V. (2015). Cloning and validation of reference genes for normalization of gene expression studies in pearl millet [*Pennisetum glaucum* (L.) R. Br.] by quantitative real-time PCR. Plant Gene.

[68] Livak K.J., Schmittgen T.D. (2001). Analysis of relative gene expression data using real-time quantitative PCR and the 2(-Delta Delta C(T)) Method. Methods.

