# Supplementary material for: Genome-Wide Identification and Transcriptional Analysis of AP2/ERF Gene Family in Pearl Millet (Pennisetum glaucum)
Source: Int J Mol Sci. 2024 Feb 20;25(5):2470. doi: 10.3390/ijms25052470 (PMC10930990; doi:10.3390/ijms25052470)
Supplement: Supplementary file 1 [file ijms-25-02470-s001.zip › Supplemental figures revised.pdf]

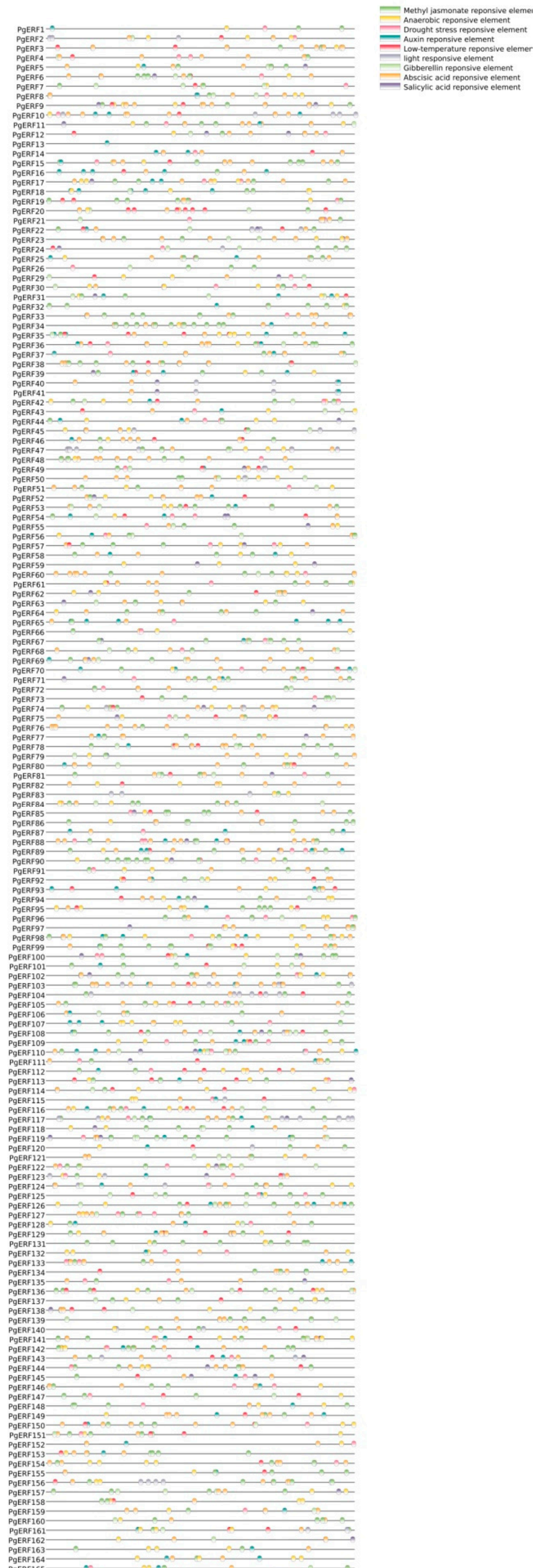

PgERF1
PgERF2
PgERF3
PgERF4
PgERF5
PgERF6
PgERF7
PgERF8
PgERF9
PgERF10
PgERF11
PgERF12
PgERF13
PgERF14
PgERF15
PgERF16
PgERF17
PgERF18
PgERF19
PgERF20
PgERF21
PgERF22
PgERF23
PgERF24
PgERF25
PgERF26
PgERF29
PgERF30
PgERF31
PgERF32
PgERF33
PgERF34
PgERF35
PgERF36
PgERF37
PgERF38
PgERF39
PgERF40
PgERF41
PgERF42
PgERF43
PgERF44
PgERF45
PgERF46
PgERF47
PgERF48
PgERF49
PgERF50
PgERF51
PgERF52
PgERF53
PgERF54
PgERF55
PgERF56
PgERF57
PgERF58
PgERF59
PgERF60
PgERF61
PgERF62
PgERF63
PgERF64
PgERF65
PgERF66
PgERF67
PgERF68
PgERF69
PgERF70
PgERF71
PgERF72
PgERF73
PgERF74
PgERF75
PgERF76
PgERF77
PgERF78
PgERF79
PgERF80
PgERF81
PgERF82
PgERF83
PgERF84
PgERF85
PgERF86
PgERF87
PgERF88
PgERF89
PgERF90
PgERF91
PgERF92
PgERF93
PgERF94
PgERF95
PgERF96
PgERF97
PgERF98
PgERF99
PgERF100
PgERF101
PgERF102
PgERF103
PgERF104
PgERF105
PgERF106
PgERF107
PgERF108
PgERF109
PgERF110
PgERF111
PgERF112
PgERF113
PgERF114
PgERF115
PgERF116
PgERF117
PgERF118
PgERF119
PgERF120
PgERF121
PgERF122
PgERF123
PgERF124
PgERF125
PgERF126
PgERF127
PgERF128
PgERF129
PgERF130
PgERF131
PgERF132
PgERF133
PgERF134
PgERF135
PgERF136
PgERF137
PgERF138
PgERF139
PgERF140
PgERF141
PgERF142
PgERF143
PgERF144
PgERF145
PgERF146
PgERF147
PgERF148
PgERF149
PgERF150
PgERF151
PgERF152
PgERF153
PgERF154
PgERF155
PgERF156
PgERF157
PgERF158
PgERF159
PgERF160
PgERF161
PgERF162
PgERF163
PgERF164
PgERF165
PgERF166
PgERF167
5'
3'
0
200
400
600
800
1000
1200
1400
1600
1800
2000
Methyl jasmonate reponsive elemen
Anaerobic reponsive element
Drought stress reponsive element
Auxin reponsive element
Low-temperature reponsive elemen
light responsive element
Gibberellin reponsive element
Abscisic acid reponsive element
Salicylic acid reponsive element

Figure S1. Promoter analysis of 167 *PgERF* genes. Abiotic stress-related *cis* elements are predicted using PlantCARE program (http://bioinformatics.psb.ugent.be/webtools/plantcare/html/, accessed on July 2023).

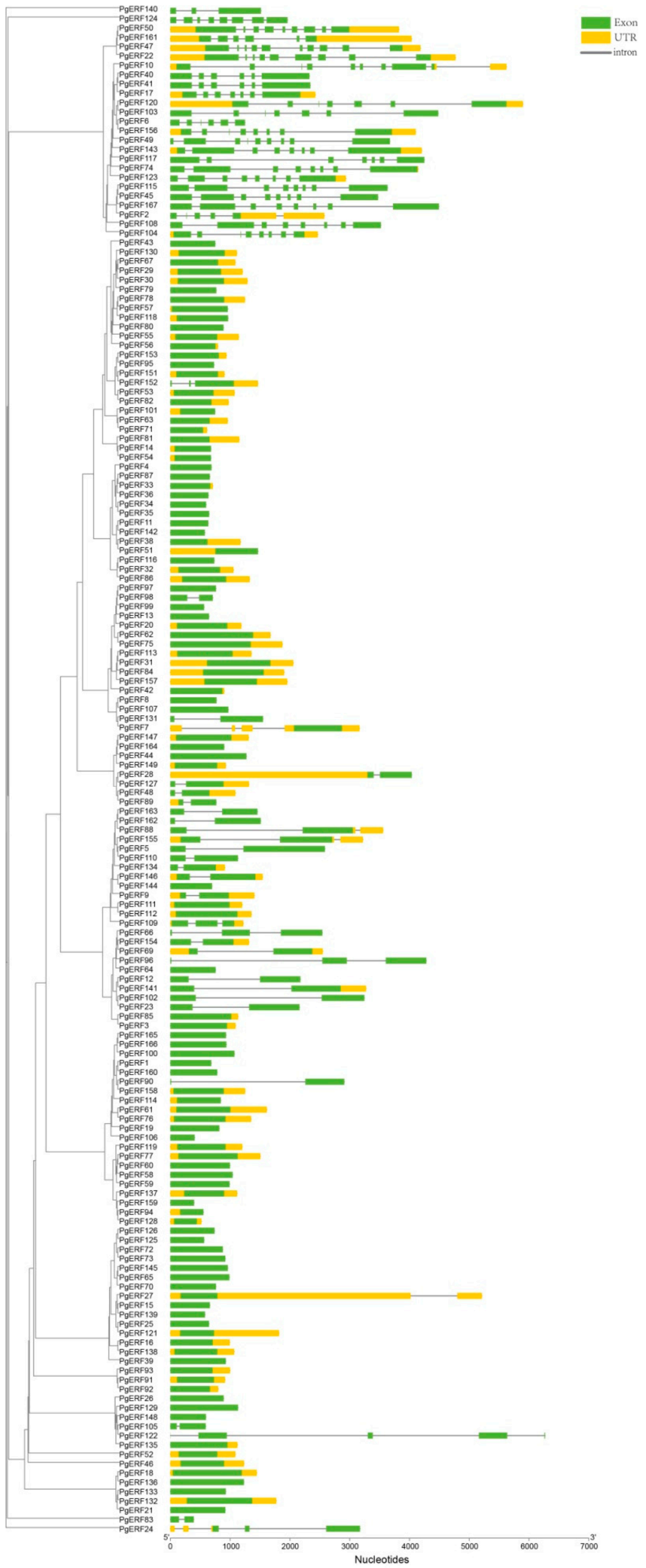
Exon
UTR
intron
PgERF140
PgERF124
PgERF50
PgERF161
PgERF47
PgERF22
PgERF10
PgERF40
PgERF41
PgERF17
PgERF120
PgERF103
PgERF6
PgERF156
PgERF49
PgERF143
PgERF117
PgERF74
PgERF123
PgERF115
PgERF45
PgERF167
PgERF2
PgERF108
PgERF104
PgERF43
PgERF130
PgERF67
PgERF29
PgERF30
PgERF79
PgERF78
PgERF57
PgERF118
PgERF80
PgERF55
PgERF56
PgERF153
PgERF95
PgERF151
PgERF152
PgERF53
PgERF82
PgERF101
PgERF63
PgERF71
PgERF81
PgERF14
PgERF54
PgERF4
PgERF87
PgERF33
PgERF36
PgERF34
PgERF35
PgERF11
PgERF142
PgERF38
PgERF51
PgERF116
PgERF32
PgERF86
PgERF97
PgERF98
PgERF99
PgERF13
PgERF20
PgERF62
PgERF75
PgERF113
PgERF31
PgERF84
PgERF157
PgERF42
PgERF8
PgERF107
PgERF131
PgERF7
PgERF147
PgERF164
PgERF44
PgERF149
PgERF28
PgERF127
PgERF48
PgERF89
PgERF163
PgERF162
PgERF88
PgERF155
PgERF5
PgERF110
PgERF134
PgERF146
PgERF144
PgERF9
PgERF111
PgERF112
PgERF109
PgERF66
PgERF154
PgERF69
PgERF96
PgERF64
PgERF12
PgERF141
PgERF102
PgERF23
PgERF85
PgERF3
PgERF165
PgERF166
PgERF100
PgERF1
PgERF160
PgERF90
PgERF158
PgERF114
PgERF61
PgERF76
PgERF19
PgERF106
PgERF119
PgERF77
PgERF60
PgERF58
PgERF59
PgERF137
PgERF159
PgERF94
PgERF128
PgERF126
PgERF125
PgERF72
PgERF73
PgERF145
PgERF65
PgERF70
PgERF27
PgERF15
PgERF139
PgERF25
PgERF121
PgERF16
PgERF138
PgERF39
PgERF93
PgERF91
PgERF92
PgERF26
PgERF129
PgERF148
PgERF105
PgERF122
PgERF135
PgERF52
PgERF46
PgERF18
PgERF136
PgERF133
PgERF132
PgERF21
PgERF83
PgERF24
5'
3'
0
1000
2000
3000
4000
5000
6000
7000
Nucleotides

Figure S2. Gene structural analysis of *PgERF* gene family. Exons, 5' and 3' Untranslated Regions (UTRs), and introns are represented by green and orange rectangle boxes, and black lines, respectively.
